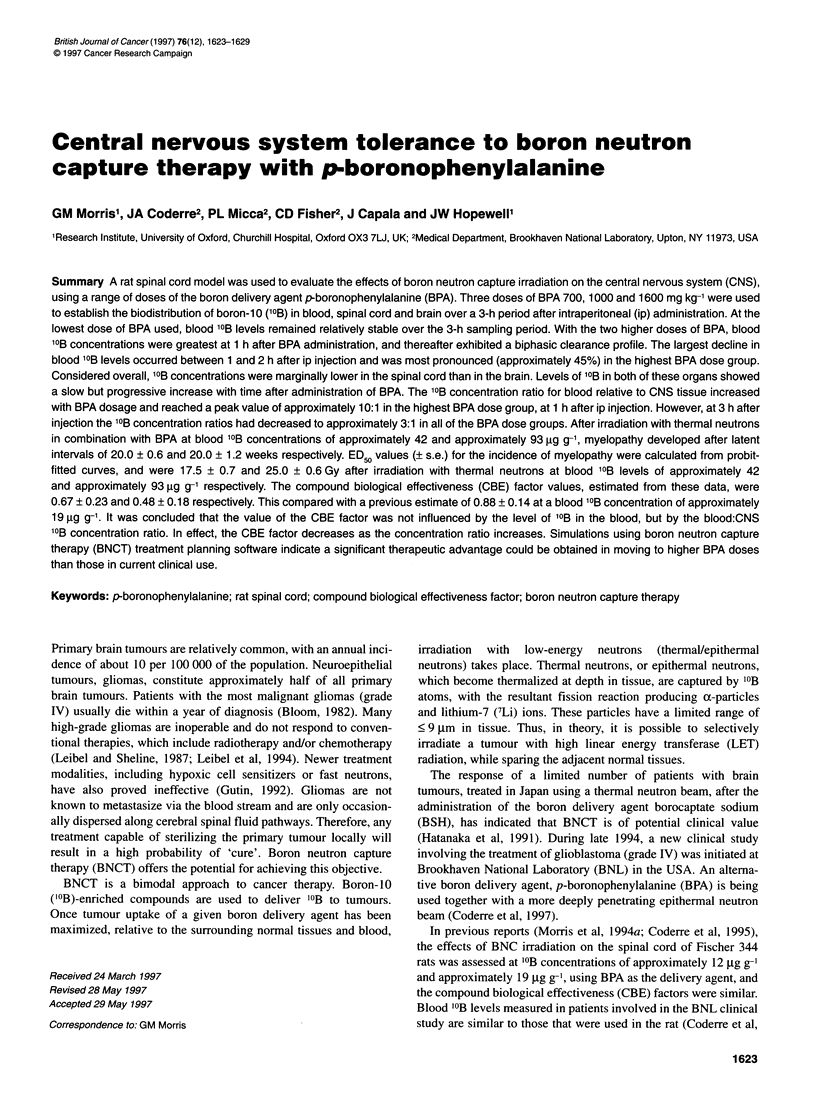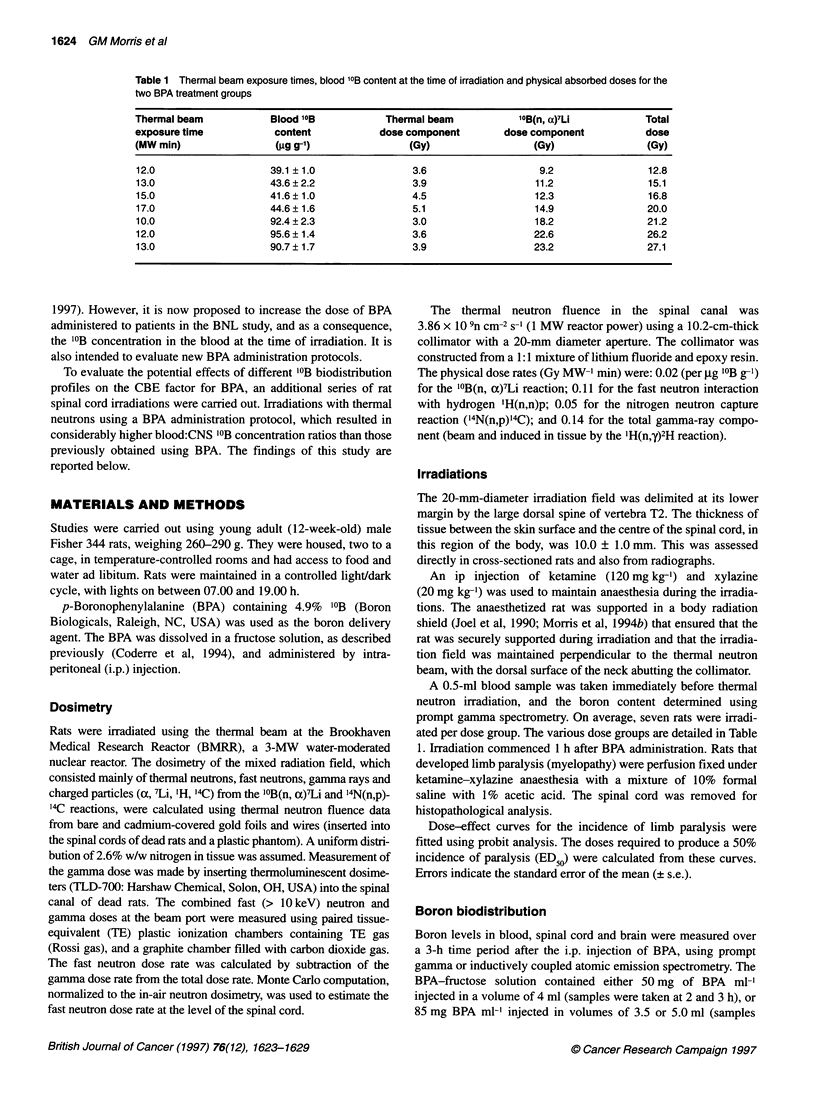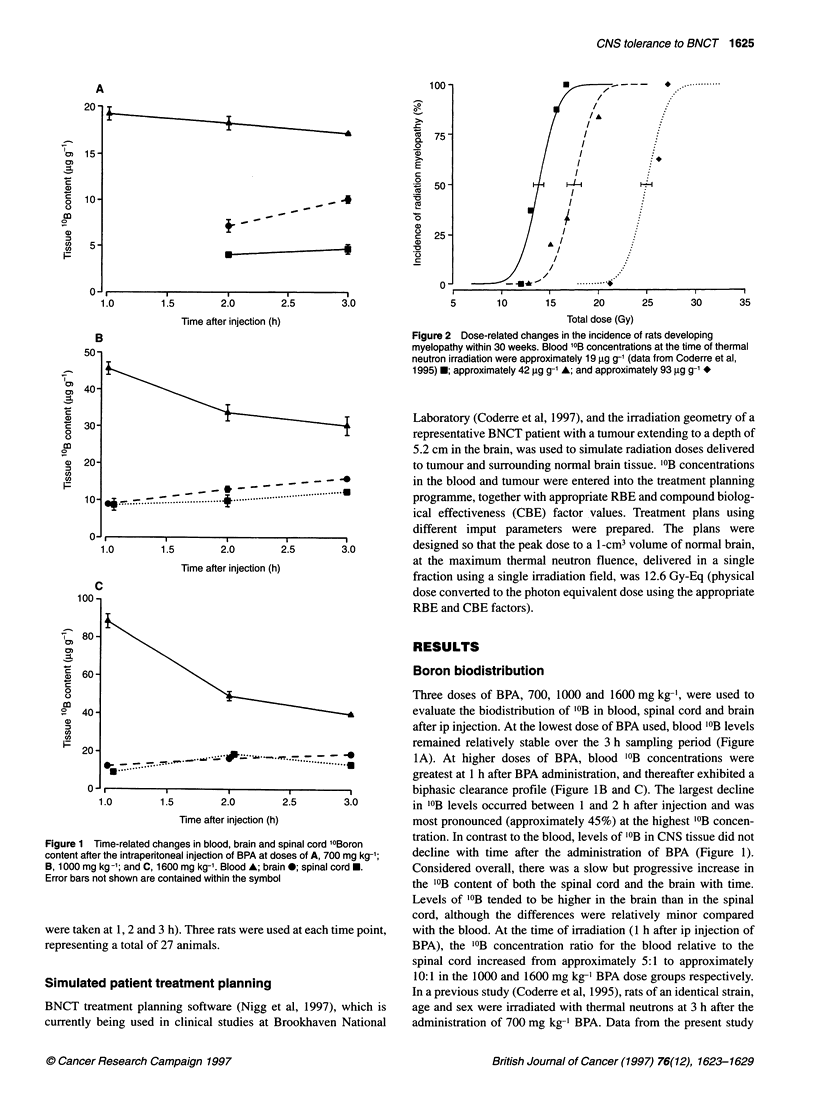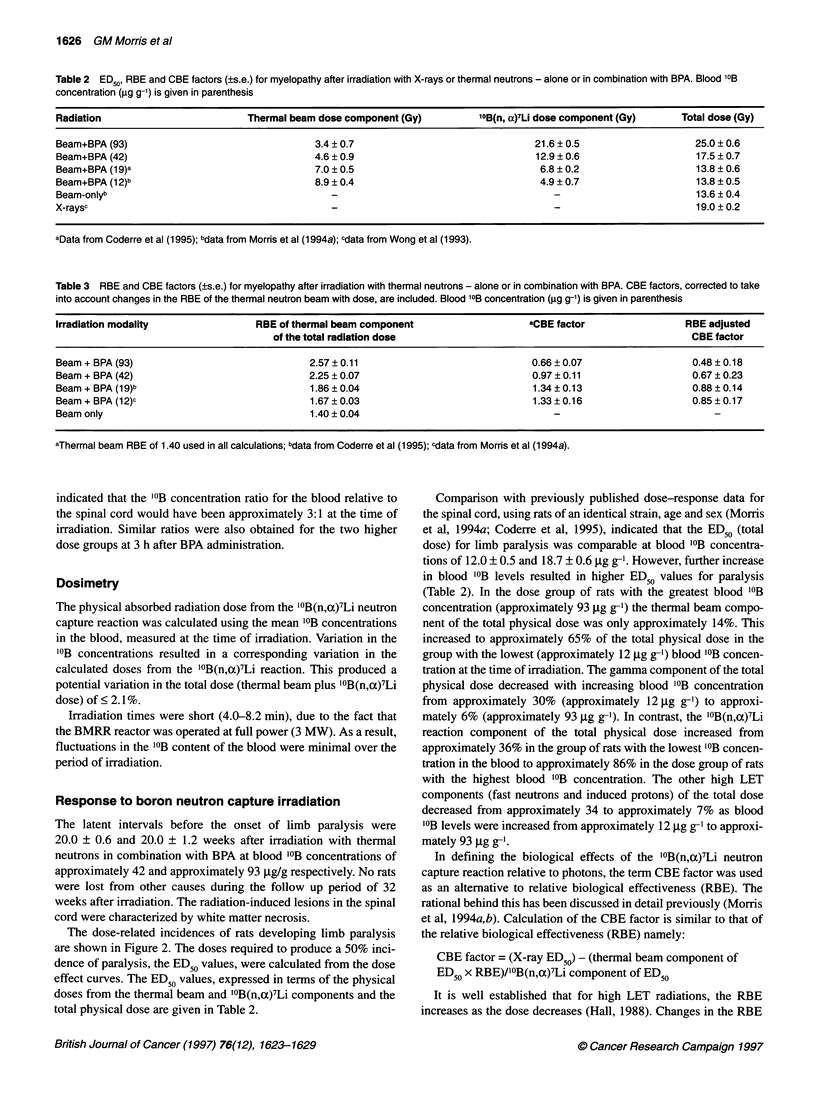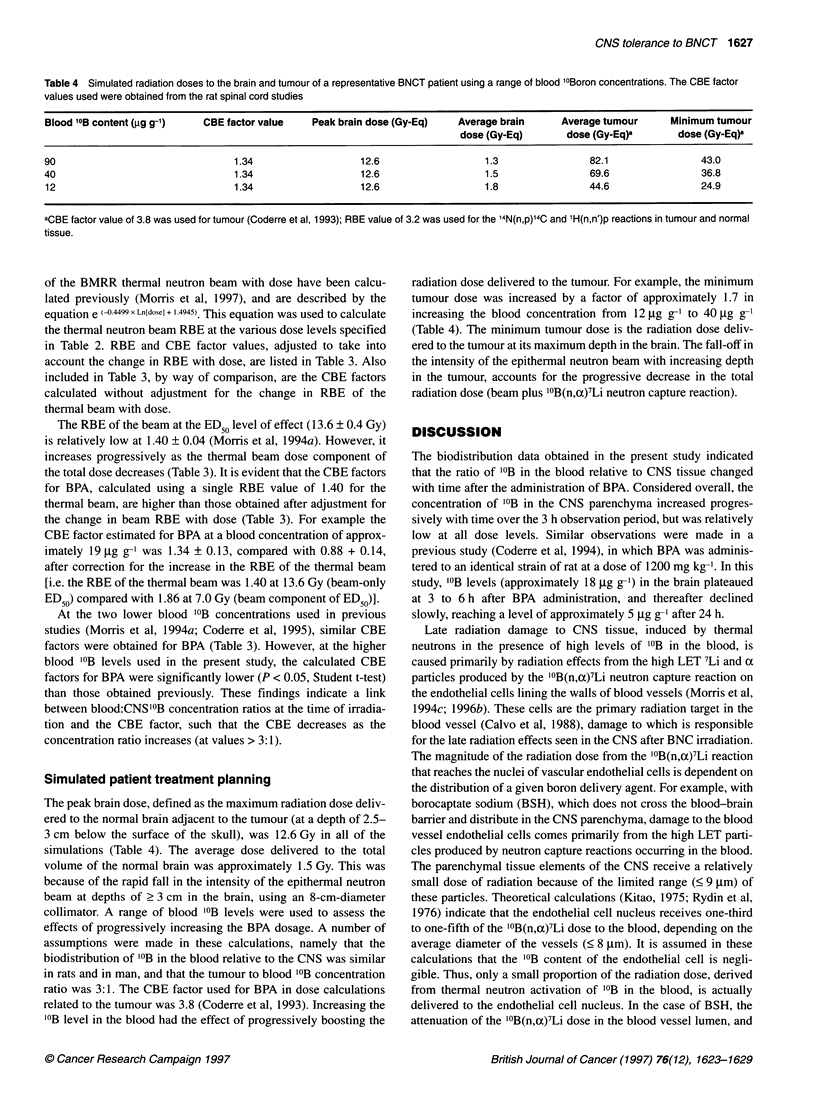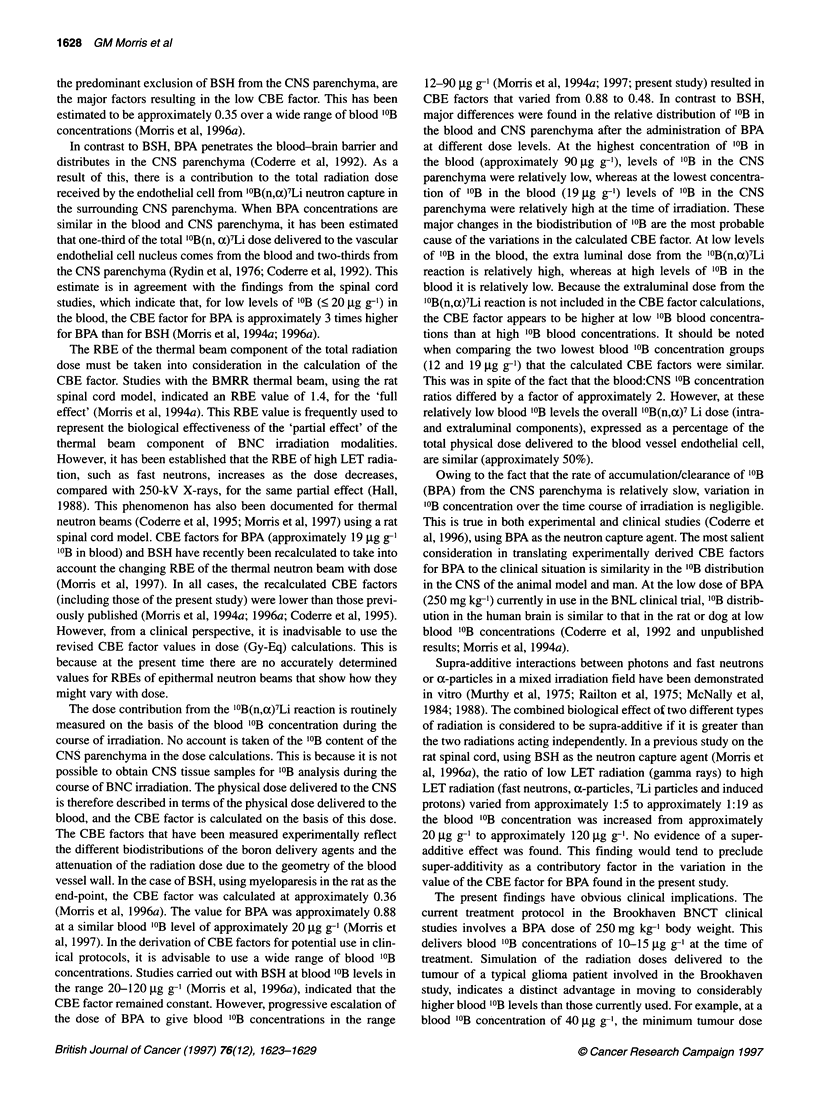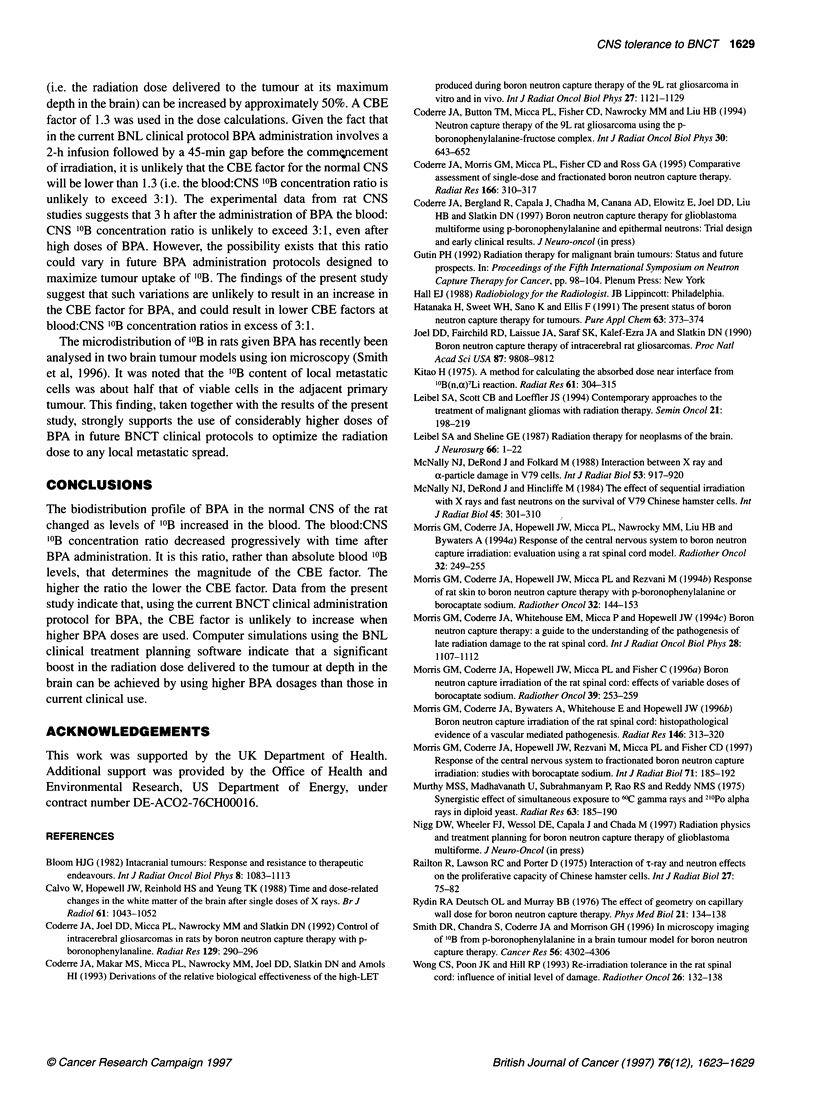# Central nervous system tolerance to boron neutron capture therapy with p-boronophenylalanine.

**DOI:** 10.1038/bjc.1997.607

**Published:** 1997

**Authors:** G. M. Morris, J. A. Coderre, P. L. Micca, C. D. Fisher, J. Capala, J. W. Hopewell

**Affiliations:** Research Institute, University of Oxford, Churchill Hospital, UK.

## Abstract

A rat spinal cord model was used to evaluate the effects of boron neutron capture irradiation on the central nervous system (CNS), using a range of doses of the boron delivery agent p-boronophenylalanine (BPA). Three doses of BPA 700, 1000 and 1600 mg kg(-1) were used to establish the biodistribution of boron-10 (10B) in blood, spinal cord and brain over a 3-h period after intraperitoneal (i.p.) administration. At the lowest dose of BPA used, blood 10B levels remained relatively stable over the 3-h sampling period. With the two higher doses of BPA, blood 10B concentrations were greatest at 1 h after BPA administration, and thereafter exhibited a biphasic clearance profile. The largest decline in blood 10B levels occurred between 1 and 2 h after i.p. injection and was most pronounced (approximately 45%) in the highest BPA dose group. Considered overall, 10B concentrations were marginally lower in the spinal cord than in the brain. Levels of 10B in both of these organs showed a slow but progressive increase with time after administration of BPA. The 10B concentration ratio for blood relative to CNS tissue increased with BPA dosage and reached a peak value of approximately 10:1 in the highest BPA dose group, at 1 h after i.p. injection. However, at 3 h after injection the 10B concentration ratios had decreased to approximately 3:1 in all of the BPA dose groups. After irradiation with thermal neutrons in combination with BPA at blood 10B concentrations of approximately 42 and approximately 93 microg g(-1), myelopathy developed after latent intervals of 20.0 +/- 0.6 and 20.0 +/- 1.2 weeks respectively. ED50 values (+/- s.e.) for the incidence of myelopathy were calculated from probit-fitted curves, and were 17.5 +/- 0.7 and 25.0 +/- 0.6 Gy after irradiation with thermal neutrons at blood 10B levels of approximately 42 and approximately 93 microg g(-1) respectively. The compound biological effectiveness (CBE) factor values, estimated from these data, were 0.67 +/- 0.23 and 0.48 +/- 0.18 respectively. This compared with a previous estimate of 0.88 +/- 0.14 at a blood 10B concentration of approximately 19 microg g(-1). It was concluded that the value of the CBE factor was not influenced by the level of 10B in the blood, but by the blood:CNS 10B concentration ratio. In effect, the CBE factor decreases as the concentration ratio increases. Simulations using boron neutron capture therapy (BNCT) treatment planning software indicate a significant therapeutic advantage could be obtained in moving to higher BPA doses than those in current clinical use.


British Journal of Cancer (1997) 76(12), 1623–1629
© 1997 Cancer Research Campaign

### Central nervous system tolerance to boron neutron capture therapy with *p*-boronophenylalanine

**GM Morris[1], JA Coderre[2], PL Micca[2], CD Fisher[2], J Capala and JW Hopewell[1]**

[1]Research Institute, University of Oxford, Churchill Hospital, Oxford OX3 7LJ, UK; [2]Medical Department, Brookhaven National Laboratory, Upton, NY 11973, USA

**Summary** A rat spinal cord model was used to evaluate the effects of boron neutron capture irradiation on the central nervous system (CNS), using a range of doses of the boron delivery agent *p*-boronophenylalanine (BPA). Three doses of BPA 700, 1000 and 1600 mg kg$^{-1}$ were used to establish the biodistribution of boron-10 ($^{10}$B) in blood, spinal cord and brain over a 3-h period after intraperitoneal (ip) administration. At the lowest dose of BPA used, blood $^{10}$B levels remained relatively stable over the 3-h sampling period. With the two higher doses of BPA, blood $^{10}$B concentrations were greatest at 1 h after BPA administration, and thereafter exhibited a biphasic clearance profile. The largest decline in blood $^{10}$B levels occurred between 1 and 2 h after ip injection and was most pronounced (approximately 45%) in the highest BPA dose group. Considered overall, $^{10}$B concentrations were marginally lower in the spinal cord than in the brain. Levels of $^{10}$B in both of these organs showed a slow but progressive increase with time after administration of BPA. The $^{10}$B concentration ratio for blood relative to CNS tissue increased with BPA dosage and reached a peak value of approximately 10:1 in the highest BPA dose group, at 1 h after ip injection. However, at 3 h after injection the $^{10}$B concentration ratios had decreased to approximately 3:1 in all of the BPA dose groups. After irradiation with thermal neutrons in combination with BPA at blood $^{10}$B concentrations of approximately 42 and approximately 93 μg g$^{-1}$, myelopathy developed after latent intervals of 20.0 ± 0.6 and 20.0 ± 1.2 weeks respectively. $ED_{50}$ values (± s.e.) for the incidence of myelopathy were calculated from probit-fitted curves, and were 17.5 ± 0.7 and 25.0 ± 0.6 Gy after irradiation with thermal neutrons at blood $^{10}$B levels of approximately 42 and approximately 93 μg g$^{-1}$ respectively. The compound biological effectiveness (CBE) factor values, estimated from these data, were 0.67 ± 0.23 and 0.48 ± 0.18 respectively. This compared with a previous estimate of 0.88 ± 0.14 at a blood $^{10}$B concentration of approximately 19 μg g$^{-1}$. It was concluded that the value of the CBE factor was not influenced by the level of $^{10}$B in the blood, but by the blood:CNS $^{10}$B concentration ratio. In effect, the CBE factor decreases as the concentration ratio increases. Simulations using boron neutron capture therapy (BNCT) treatment planning software indicate a significant therapeutic advantage could be obtained in moving to higher BPA doses than those in current clinical use.

**Keywords:** *p*-boronophenylalanine; rat spinal cord; compound biological effectiveness factor; boron neutron capture therapy

Primary brain tumours are relatively common, with an annual incidence of about 10 per 100 000 of the population. Neuroepithelial tumours, gliomas, constitute approximately half of all primary brain tumours. Patients with the most malignant gliomas (grade IV) usually die within a year of diagnosis (Bloom, 1982). Many high-grade gliomas are inoperable and do not respond to conventional therapies, which include radiotherapy and/or chemotherapy (Leibel and Sheline, 1987; Leibel et al, 1994). Newer treatment modalities, including hypoxic cell sensitizers or fast neutrons, have also proved ineffective (Gutin, 1992). Gliomas are not known to metastasize via the blood stream and are only occasionally dispersed along cerebral spinal fluid pathways. Therefore, any treatment capable of sterilizing the primary tumour locally will result in a high probability of 'cure'. Boron neutron capture therapy (BNCT) offers the potential for achieving this objective.

BNCT is a bimodal approach to cancer therapy. Boron-10 ($^{10}$B)-enriched compounds are used to deliver $^{10}$B to tumours. Once tumour uptake of a given boron delivery agent has been maximized, relative to the surrounding normal tissues and blood, irradiation with low-energy neutrons (thermal/epithermal neutrons) takes place. Thermal neutrons, or epithermal neutrons, which become thermalized at depth in tissue, are captured by $^{10}$B atoms, with the resultant fission reaction producing α-particles and lithium-7 ($^{7}$Li) ions. These particles have a limited range of ≤ 9 μm in tissue. Thus, in theory, it is possible to selectively irradiate a tumour with high linear energy transferase (LET) radiation, while sparing the adjacent normal tissues.

The response of a limited number of patients with brain tumours, treated in Japan using a thermal neutron beam, after the administration of the boron delivery agent borocaptate sodium (BSH), has indicated that BNCT is of potential clinical value (Hatanaka et al, 1991). During late 1994, a new clinical study involving the treatment of glioblastoma (grade IV) was initiated at Brookhaven National Laboratory (BNL) in the USA. An alternative boron delivery agent, *p*-boronophenylalanine (BPA) is being used together with a more deeply penetrating epithermal neutron beam (Coderre et al, 1997).

In previous reports (Morris et al, 1994*a*; Coderre et al, 1995), the effects of BNC irradiation on the spinal cord of Fischer 344 rats was assessed at $^{10}$B concentrations of approximately 12 μg g$^{-1}$ and approximately 19 μg g$^{-1}$, using BPA as the delivery agent, and the compound biological effectiveness (CBE) factors were similar. Blood $^{10}$B levels measured in patients involved in the BNL clinical study are similar to those that were used in the rat (Coderre et al,

*Received 24 March 1997*
*Revised 28 May 1997*
*Accepted 29 May 1997*

*Correspondence to:* GM Morris

**Table 1** Thermal beam exposure times, blood $^{10}B$ content at the time of irradiation and physical absorbed doses for the two BPA treatment groups

| Thermal beam exposure time (MW min) | Blood $^{10}B$ content ($\mu g\ g^{-1}$) | Thermal beam dose component (Gy) | $^{10}B(n, \alpha)^7Li$ dose component (Gy) | Total dose (Gy) |
|---|---|---|---|---|
| 12.0 | 39.1 ± 1.0 | 3.6 | 9.2 | 12.8 |
| 13.0 | 43.6 ± 2.2 | 3.9 | 11.2 | 15.1 |
| 15.0 | 41.6 ± 1.0 | 4.5 | 12.3 | 16.8 |
| 17.0 | 44.6 ± 1.6 | 5.1 | 14.9 | 20.0 |
| 10.0 | 92.4 ± 2.3 | 3.0 | 18.2 | 21.2 |
| 12.0 | 95.6 ± 1.4 | 3.6 | 22.6 | 26.2 |
| 13.0 | 90.7 ± 1.7 | 3.9 | 23.2 | 27.1 |

1997). However, it is now proposed to increase the dose of BPA administered to patients in the BNL study, and as a consequence, the $^{10}B$ concentration in the blood at the time of irradiation. It is also intended to evaluate new BPA administration protocols.

To evaluate the potential effects of different $^{10}B$ biodistribution profiles on the CBE factor for BPA, an additional series of rat spinal cord irradiations were carried out. Irradiations with thermal neutrons using a BPA administration protocol, which resulted in considerably higher blood:CNS $^{10}B$ concentration ratios than those previously obtained using BPA. The findings of this study are reported below.

### MATERIALS AND METHODS

Studies were carried out using young adult (12-week-old) male Fisher 344 rats, weighing 260–290 g. They were housed, two to a cage, in temperature-controlled rooms and had access to food and water ad libitum. Rats were maintained in a controlled light/dark cycle, with lights on between 07.00 and 19.00 h.

*p*-Boronophenylalanine (BPA) containing 4.9% $^{10}B$ (Boron Biologicals, Raleigh, NC, USA) was used as the boron delivery agent. The BPA was dissolved in a fructose solution, as described previously (Coderre et al, 1994), and administered by intraperitoneal (i.p.) injection.

### Dosimetry

Rats were irradiated using the thermal beam at the Brookhaven Medical Research Reactor (BMRR), a 3-MW water-moderated nuclear reactor. The dosimetry of the mixed radiation field, which consisted mainly of thermal neutrons, fast neutrons, gamma rays and charged particles ($\alpha$, $^7Li$, $^1H$, $^{14}C$) from the $^{10}B(n, \alpha)^7Li$ and $^{14}N(n,p)$-$^{14}C$ reactions, were calculated using thermal neutron fluence data from bare and cadmium-covered gold foils and wires (inserted into the spinal cords of dead rats and a plastic phantom). A uniform distribution of 2.6% w/w nitrogen in tissue was assumed. Measurement of the gamma dose was made by inserting thermoluminescent dosimeters (TLD-700: Harshaw Chemical, Solon, OH, USA) into the spinal canal of dead rats. The combined fast (> 10 keV) neutron and gamma doses at the beam port were measured using paired tissue-equivalent (TE) plastic ionization chambers containing TE gas (Rossi gas), and a graphite chamber filled with carbon dioxide gas. The fast neutron dose rate was calculated by subtraction of the gamma dose rate from the total dose rate. Monte Carlo computation, normalized to the in-air neutron dosimetry, was used to estimate the fast neutron dose rate at the level of the spinal cord.

The thermal neutron fluence in the spinal canal was $3.86 \times 10^9$ n $cm^{-2}$ $s^{-1}$ (1 MW reactor power) using a 10.2-cm-thick collimator with a 20-mm diameter aperture. The collimator was constructed from a 1:1 mixture of lithium fluoride and epoxy resin. The physical dose rates (Gy $MW^{-1}$ min) were: 0.02 (per $\mu g$ $^{10}B$ $g^{-1}$) for the $^{10}B(n, \alpha)^7Li$ reaction; 0.11 for the fast neutron interaction with hydrogen $^1H(n,n)p$; 0.05 for the nitrogen neutron capture reaction ($^{14}N(n,p)^{14}C$); and 0.14 for the total gamma-ray component (beam and induced in tissue by the $^1H(n,\gamma)^2H$ reaction).

### Irradiations

The 20-mm-diameter irradiation field was delimited at its lower margin by the large dorsal spine of vertebra T2. The thickness of tissue between the skin surface and the centre of the spinal cord, in this region of the body, was 10.0 ± 1.0 mm. This was assessed directly in cross-sectioned rats and also from radiographs.

An ip injection of ketamine (120 mg $kg^{-1}$) and xylazine (20 mg $kg^{-1}$) was used to maintain anaesthesia during the irradiations. The anaesthetized rat was supported in a body radiation shield (Joel et al, 1990; Morris et al, 1994*b*) that ensured that the rat was securely supported during irradiation and that the irradiation field was maintained perpendicular to the thermal neutron beam, with the dorsal surface of the neck abutting the collimator.

A 0.5-ml blood sample was taken immediately before thermal neutron irradiation, and the boron content determined using prompt gamma spectrometry. On average, seven rats were irradiated per dose group. The various dose groups are detailed in Table 1. Irradiation commenced 1 h after BPA administration. Rats that developed limb paralysis (myelopathy) were perfusion fixed under ketamine–xylazine anaesthesia with a mixture of 10% formal saline with 1% acetic acid. The spinal cord was removed for histopathological analysis.

Dose–effect curves for the incidence of limb paralysis were fitted using probit analysis. The doses required to produce a 50% incidence of paralysis ($ED_{50}$) were calculated from these curves. Errors indicate the standard error of the mean (± s.e.).

### Boron biodistribution

Boron levels in blood, spinal cord and brain were measured over a 3-h time period after the i.p. injection of BPA, using prompt gamma or inductively coupled atomic emission spectrometry. The BPA–fructose solution contained either 50 mg of BPA $ml^{-1}$ injected in a volume of 4 ml (samples were taken at 2 and 3 h), or 85 mg BPA $ml^{-1}$ injected in volumes of 3.5 or 5.0 ml (samples

© Cancer Research Campaign 1997

**Figure 1** Time-related changes in blood, brain and spinal cord $^{10}$Boron content after the intraperitoneal injection of BPA at doses of **A**, 700 mg kg$^{-1}$; **B**, 1000 mg kg$^{-1}$; and **C**, 1600 mg kg$^{-1}$. Blood ▲; brain ●; spinal cord ■. Error bars not shown are contained within the symbol were taken at 1, 2 and 3 h). Three rats were used at each time point, representing a total of 27 animals.

### Simulated patient treatment planning

BNCT treatment planning software (Nigg et al, 1997), which is currently being used in clinical studies at Brookhaven National Laboratory (Coderre et al, 1997), and the irradiation geometry of a representative BNCT patient with a tumour extending to a depth of 5.2 cm in the brain, was used to simulate radiation doses delivered to tumour and surrounding normal brain tissue. $^{10}$B concentrations in the blood and tumour were entered into the treatment planning programme, together with appropriate RBE and compound biological effectiveness (CBE) factor values. Treatment plans using different imput parameters were prepared. The plans were designed so that the peak dose to a 1-cm$^3$ volume of normal brain, at the maximum thermal neutron fluence, delivered in a single fraction using a single irradiation field, was 12.6 Gy-Eq (physical dose converted to the photon equivalent dose using the appropriate RBE and CBE factors).

**Figure 2** Dose-related changes in the incidence of rats developing myelopathy within 30 weeks. Blood $^{10}$B concentrations at the time of thermal neutron irradiation were approximately 19 µg g$^{-1}$ (data from Coderre et al, 1995) ■; approximately 42 µg g$^{-1}$ ▲; and approximately 93 µg g$^{-1}$ ◆

### RESULTS

### Boron biodistribution

Three doses of BPA, 700, 1000 and 1600 mg kg$^{-1}$, were used to evaluate the biodistribution of $^{10}$B in blood, spinal cord and brain after ip injection. At the lowest dose of BPA used, blood $^{10}$B levels remained relatively stable over the 3 h sampling period (Figure 1A). At higher doses of BPA, blood $^{10}$B concentrations were greatest at 1 h after BPA administration, and thereafter exhibited a biphasic clearance profile (Figure 1B and C). The largest decline in $^{10}$B levels occurred between 1 and 2 h after injection and was most pronounced (approximately 45%) at the highest $^{10}$B concentration. In contrast to the blood, levels of $^{10}$B in CNS tissue did not decline with time after the administration of BPA (Figure 1). Considered overall, there was a slow but progressive increase in the $^{10}$B content of both the spinal cord and the brain with time. Levels of $^{10}$B tended to be higher in the brain than in the spinal cord, although the differences were relatively minor compared with the blood. At the time of irradiation (1 h after ip injection of BPA), the $^{10}$B concentration ratio for the blood relative to the spinal cord increased from approximately 5:1 to approximately 10:1 in the 1000 and 1600 mg kg$^{-1}$ BPA dose groups respectively. In a previous study (Coderre et al, 1995), rats of an identical strain, age and sex were irradiated with thermal neutrons at 3 h after the administration of 700 mg kg$^{-1}$ BPA. Data from the present study

**Table 2** $ED_{50}$, RBE and CBE factors (±s.e.) for myelopathy after irradiation with X-rays or thermal neutrons – alone or in combination with BPA. Blood $^{10}B$ concentration (μg $g^{-1}$) is given in parenthesis

| Radiation | Thermal beam dose component (Gy) | $^{10}B(n, \alpha)^7Li$ dose component (Gy) | Total dose (Gy) |
|---|---|---|---|
| Beam+BPA (93) | 3.4 ± 0.7 | 21.6 ± 0.5 | 25.0 ± 0.6 |
| Beam+BPA (42) | 4.6 ± 0.9 | 12.9 ± 0.6 | 17.5 ± 0.7 |
| Beam+BPA (19)[a] | 7.0 ± 0.5 | 6.8 ± 0.2 | 13.8 ± 0.6 |
| Beam+BPA (12)[b] | 8.9 ± 0.4 | 4.9 ± 0.7 | 13.8 ± 0.5 |
| Beam-only[b] | – | – | 13.6 ± 0.4 |
| X-rays[c] | – | – | 19.0 ± 0.2 |

[a]Data from Coderre et al (1995); [b]data from Morris et al (1994*a*); [c]data from Wong et al (1993).

**Table 3** RBE and CBE factors (±s.e.) for myelopathy after irradiation with thermal neutrons – alone or in combination with BPA. CBE factors, corrected to take into account changes in the RBE of the thermal neutron beam with dose, are included. Blood $^{10}B$ concentration (μg $g^{-1}$) is given in parenthesis

| Irradiation modality | RBE of thermal beam component of the total radiation dose | [a]CBE factor | RBE adjusted CBE factor |
|---|---|---|---|
| Beam + BPA (93) | 2.57 ± 0.11 | 0.66 ± 0.07 | 0.48 ± 0.18 |
| Beam + BPA (42) | 2.25 ± 0.07 | 0.97 ± 0.11 | 0.67 ± 0.23 |
| Beam + BPA (19)[b] | 1.86 ± 0.04 | 1.34 ± 0.13 | 0.88 ± 0.14 |
| Beam + BPA (12)[c] | 1.67 ± 0.03 | 1.33 ± 0.16 | 0.85 ± 0.17 |
| Beam only | 1.40 ± 0.04 | – | – |

[a]Thermal beam RBE of 1.40 used in all calculations; [b]data from Coderre et al (1995); [c]data from Morris et al (1994*a*).

indicated that the $^{10}B$ concentration ratio for the blood relative to the spinal cord would have been approximately 3:1 at the time of irradiation. Similar ratios were also obtained for the two higher dose groups at 3 h after BPA administration.

### Dosimetry

The physical absorbed radiation dose from the $^{10}B(n,\alpha)^7Li$ neutron capture reaction was calculated using the mean $^{10}B$ concentrations in the blood, measured at the time of irradiation. Variation in the $^{10}B$ concentrations resulted in a corresponding variation in the calculated doses from the $^{10}B(n,\alpha)^7Li$ reaction. This produced a potential variation in the total dose (thermal beam plus $^{10}B(n,\alpha)^7Li$ dose) of ≤ 2.1%.

Irradiation times were short (4.0–8.2 min), due to the fact that the BMRR reactor was operated at full power (3 MW). As a result, fluctuations in the $^{10}B$ content of the blood were minimal over the period of irradiation.

### Response to boron neutron capture irradiation

The latent intervals before the onset of limb paralysis were 20.0 ± 0.6 and 20.0 ± 1.2 weeks after irradiation with thermal neutrons in combination with BPA at blood $^{10}B$ concentrations of approximately 42 and approximately 93 μg/g respectively. No rats were lost from other causes during the follow up period of 32 weeks after irradiation. The radiation-induced lesions in the spinal cord were characterized by white matter necrosis.

The dose-related incidences of rats developing limb paralysis are shown in Figure 2. The doses required to produce a 50% incidence of paralysis, the $ED_{50}$ values, were calculated from the dose effect curves. The $ED_{50}$ values, expressed in terms of the physical doses from the thermal beam and $^{10}B(n,\alpha)^7Li$ components and the total physical dose are given in Table 2.

Comparison with previously published dose–response data for the spinal cord, using rats of an identical strain, age and sex (Morris et al, 1994*a*; Coderre et al, 1995), indicated that the $ED_{50}$ (total dose) for limb paralysis was comparable at blood $^{10}B$ concentrations of 12.0 ± 0.5 and 18.7 ± 0.6 μg $g^{-1}$. However, further increase in blood $^{10}B$ levels resulted in higher $ED_{50}$ values for paralysis (Table 2). In the dose group of rats with the greatest blood $^{10}B$ concentration (approximately 93 μg $g^{-1}$) the thermal beam component of the total physical dose was only approximately 14%. This increased to approximately 65% of the total physical dose in the group with the lowest (approximately 12 μg $g^{-1}$) blood $^{10}B$ concentration at the time of irradiation. The gamma component of the total physical dose decreased with increasing blood $^{10}B$ concentration from approximately 30% (approximately 12 μg $g^{-1}$) to approximately 6% (approximately 93 μg $g^{-1}$). In contrast, the $^{10}B(n,\alpha)^7Li$ reaction component of the total physical dose increased from approximately 36% in the group of rats with the lowest $^{10}B$ concentration in the blood to approximately 86% in the dose group of rats with the highest blood $^{10}B$ concentration. The other high LET components (fast neutrons and induced protons) of the total dose decreased from approximately 34 to approximately 7% as blood $^{10}B$ levels were increased from approximately 12 μg $g^{-1}$ to approximately 93 μg $g^{-1}$.

In defining the biological effects of the $^{10}B(n,\alpha)^7Li$ neutron capture reaction relative to photons, the term CBE factor was used as an alternative to relative biological effectiveness (RBE). The rational behind this has been discussed in detail previously (Morris et al, 1994*a,b*). Calculation of the CBE factor is similar to that of the relative biological effectiveness (RBE) namely:

$$\text{CBE factor} = (\text{X-ray } ED_{50}) - (\text{thermal beam component of } ED_{50} \times \text{RBE}) / {}^{10}B(n,\alpha)^7Li \text{ component of } ED_{50}$$

It is well established that for high LET radiations, the RBE increases as the dose decreases (Hall, 1988). Changes in the RBE

© Cancer Research Campaign 1997

**Table 4** Simulated radiation doses to the brain and tumour of a representative BNCT patient using a range of blood $^{10}$Boron concentrations. The CBE factor values used were obtained from the rat spinal cord studies

| Blood $^{10}$B content (μg g$^{-1}$) | CBE factor value | Peak brain dose (Gy-Eq) | Average brain dose (Gy-Eq) | Average tumour dose (Gy-Eq)[a] | Minimum tumour dose (Gy-Eq)[a] |
|---|---|---|---|---|---|
| 90 | 1.34 | 12.6 | 1.3 | 82.1 | 43.0 |
| 40 | 1.34 | 12.6 | 1.5 | 69.6 | 36.8 |
| 12 | 1.34 | 12.6 | 1.8 | 44.6 | 24.9 |

[a]CBE factor value of 3.8 was used for tumour (Coderre et al, 1993); RBE value of 3.2 was used for the $^{14}N(n,p)^{14}C$ and $^{1}H(n,n')p$ reactions in tumour and normal tissue.

of the BMRR thermal neutron beam with dose have been calculated previously (Morris et al, 1997), and are described by the equation $e^{(-0.4499 \times \mathrm{Ln[dose]} + 1.4945)}$. This equation was used to calculate the thermal neutron beam RBE at the various dose levels specified in Table 2. RBE and CBE factor values, adjusted to take into account the change in RBE with dose, are listed in Table 3. Also included in Table 3, by way of comparison, are the CBE factors calculated without adjustment for the change in RBE of the thermal beam with dose.

The RBE of the beam at the $ED_{50}$ level of effect (13.6 ± 0.4 Gy) is relatively low at 1.40 ± 0.04 (Morris et al, 1994*a*). However, it increases progressively as the thermal beam dose component of the total dose decreases (Table 3). It is evident that the CBE factors for BPA, calculated using a single RBE value of 1.40 for the thermal beam, are higher than those obtained after adjustment for the change in beam RBE with dose (Table 3). For example the CBE factor estimated for BPA at a blood concentration of approximately 19 μg g$^{-1}$ was 1.34 ± 0.13, compared with 0.88 + 0.14, after correction for the increase in the RBE of the thermal beam [i.e. the RBE of the thermal beam was 1.40 at 13.6 Gy (beam-only $ED_{50}$) compared with 1.86 at 7.0 Gy (beam component of $ED_{50}$)].

At the two lower blood $^{10}$B concentrations used in previous studies (Morris et al, 1994*a*; Coderre et al, 1995), similar CBE factors were obtained for BPA (Table 3). However, at the higher blood $^{10}$B levels used in the present study, the calculated CBE factors for BPA were significantly lower ($P < 0.05$, Student t-test) than those obtained previously. These findings indicate a link between blood:CNS $^{10}$B concentration ratios at the time of irradiation and the CBE factor, such that the CBE decreases as the concentration ratio increases (at values > 3:1).

### Simulated patient treatment planning

The peak brain dose, defined as the maximum radiation dose delivered to the normal brain adjacent to the tumour (at a depth of 2.5–3 cm below the surface of the skull), was 12.6 Gy in all of the simulations (Table 4). The average dose delivered to the total volume of the normal brain was approximately 1.5 Gy. This was because of the rapid fall in the intensity of the epithermal neutron beam at depths of ≥ 3 cm in the brain, using an 8-cm-diameter collimator. A range of blood $^{10}$B levels were used to assess the effects of progressively increasing the BPA dosage. A number of assumptions were made in these calculations, namely that the biodistribution of $^{10}$B in the blood relative to the CNS was similar in rats and in man, and that the tumour to blood $^{10}$B concentration ratio was 3:1. The CBE factor used for BPA in dose calculations related to the tumour was 3.8 (Coderre et al, 1993). Increasing the $^{10}$B level in the blood had the effect of progressively boosting the radiation dose delivered to the tumour. For example, the minimum tumour dose was increased by a factor of approximately 1.7 in increasing the blood concentration from 12 μg g$^{-1}$ to 40 μg g$^{-1}$ (Table 4). The minimum tumour dose is the radiation dose delivered to the tumour at its maximum depth in the brain. The fall-off in the intensity of the epithermal neutron beam with increasing depth in the tumour, accounts for the progressive decrease in the total radiation dose (beam plus $^{10}B(n,\alpha)^{7}Li$ neutron capture reaction).

### DISCUSSION

The biodistribution data obtained in the present study indicated that the ratio of $^{10}$B in the blood relative to CNS tissue changed with time after the administration of BPA. Considered overall, the concentration of $^{10}$B in the CNS parenchyma increased progressively with time over the 3 h observation period, but was relatively low at all dose levels. Similar observations were made in a previous study (Coderre et al, 1994), in which BPA was administered to an identical strain of rat at a dose of 1200 mg kg$^{-1}$. In this study, $^{10}$B levels (approximately 18 μg g$^{-1}$) in the brain plateaued at 3 to 6 h after BPA administration, and thereafter declined slowly, reaching a level of approximately 5 μg g$^{-1}$ after 24 h.

Late radiation damage to CNS tissue, induced by thermal neutrons in the presence of high levels of $^{10}$B in the blood, is caused primarily by radiation effects from the high LET $^{7}$Li and α particles produced by the $^{10}B(n,\alpha)^{7}Li$ neutron capture reaction on the endothelial cells lining the walls of blood vessels (Morris et al, 1994*c*; 1996*b*). These cells are the primary radiation target in the blood vessel (Calvo et al, 1988), damage to which is responsible for the late radiation effects seen in the CNS after BNC irradiation. The magnitude of the radiation dose from the $^{10}B(n,\alpha)^{7}Li$ reaction that reaches the nuclei of vascular endothelial cells is dependent on the distribution of a given boron delivery agent. For example, with borocaptate sodium (BSH), which does not cross the blood–brain barrier and distribute in the CNS parenchyma, damage to the blood vessel endothelial cells comes primarily from the high LET particles produced by neutron capture reactions occurring in the blood. The parenchymal tissue elements of the CNS receive a relatively small dose of radiation because of the limited range (≤ 9 μm) of these particles. Theoretical calculations (Kitao, 1975; Rydin et al, 1976) indicate that the endothelial cell nucleus receives one-third to one-fifth of the $^{10}B(n,\alpha)^{7}Li$ dose to the blood, depending on the average diameter of the vessels (≤ 8 μm). It is assumed in these calculations that the $^{10}$B content of the endothelial cell is negligible. Thus, only a small proportion of the radiation dose, derived from thermal neutron activation of $^{10}$B in the blood, is actually delivered to the endothelial cell nucleus. In the case of BSH, the attenuation of the $^{10}B(n,\alpha)^{7}Li$ dose in the blood vessel lumen, and the predominant exclusion of BSH from the CNS parenchyma, are the major factors resulting in the low CBE factor. This has been estimated to be approximately 0.35 over a wide range of blood $^{10}B$ concentrations (Morris et al, 1996*a*).

In contrast to BSH, BPA penetrates the blood–brain barrier and distributes in the CNS parenchyma (Coderre et al, 1992). As a result of this, there is a contribution to the total radiation dose received by the endothelial cell from $^{10}B(n,\alpha)^7Li$ neutron capture in the surrounding CNS parenchyma. When BPA concentrations are similar in the blood and CNS parenchyma, it has been estimated that one-third of the total $^{10}B(n, \alpha)^7Li$ dose delivered to the vascular endothelial cell nucleus comes from the blood and two-thirds from the CNS parenchyma (Rydin et al, 1976; Coderre et al, 1992). This estimate is in agreement with the findings from the spinal cord studies, which indicate that, for low levels of $^{10}B$ ($\leq 20\ \mu g\ g^{-1}$) in the blood, the CBE factor for BPA is approximately 3 times higher for BPA than for BSH (Morris et al, 1994*a*; 1996*a*).

The RBE of the thermal beam component of the total radiation dose must be taken into consideration in the calculation of the CBE factor. Studies with the BMRR thermal beam, using the rat spinal cord model, indicated an RBE value of 1.4, for the 'full effect' (Morris et al, 1994*a*). This RBE value is frequently used to represent the biological effectiveness of the 'partial effect' of the thermal beam component of BNC irradiation modalities. However, it has been established that the RBE of high LET radiation, such as fast neutrons, increases as the dose decreases, compared with 250-kV X-rays, for the same partial effect (Hall, 1988). This phenomenon has also been documented for thermal neutron beams (Coderre et al, 1995; Morris et al, 1997) using a rat spinal cord model. CBE factors for BPA (approximately 19 $\mu g\ g^{-1}$ $^{10}B$ in blood) and BSH have recently been recalculated to take into account the changing RBE of the thermal neutron beam with dose (Morris et al, 1997). In all cases, the recalculated CBE factors (including those of the present study) were lower than those previously published (Morris et al, 1994*a*; 1996*a*; Coderre et al, 1995). However, from a clinical perspective, it is inadvisable to use the revised CBE factor values in dose (Gy-Eq) calculations. This is because at the present time there are no accurately determined values for RBEs of epithermal neutron beams that show how they might vary with dose.

The dose contribution from the $^{10}B(n,\alpha)^7Li$ reaction is routinely measured on the basis of the blood $^{10}B$ concentration during the course of irradiation. No account is taken of the $^{10}B$ content of the CNS parenchyma in the dose calculations. This is because it is not possible to obtain CNS tissue samples for $^{10}B$ analysis during the course of BNC irradiation. The physical dose delivered to the CNS is therefore described in terms of the physical dose delivered to the blood, and the CBE factor is calculated on the basis of this dose. The CBE factors that have been measured experimentally reflect the different biodistributions of the boron delivery agents and the attenuation of the radiation dose due to the geometry of the blood vessel wall. In the case of BSH, using myeloparesis in the rat as the end-point, the CBE factor was calculated at approximately 0.36 (Morris et al, 1996*a*). The value for BPA was approximately 0.88 at a similar blood $^{10}B$ level of approximately 20 $\mu g\ g^{-1}$ (Morris et al, 1997). In the derivation of CBE factors for potential use in clinical protocols, it is advisable to use a wide range of blood $^{10}B$ concentrations. Studies carried out with BSH at blood $^{10}B$ levels in the range 20–120 $\mu g\ g^{-1}$ (Morris et al, 1996*a*), indicated that the CBE factor remained constant. However, progressive escalation of the dose of BPA to give blood $^{10}B$ concentrations in the range 12–90 $\mu g\ g^{-1}$ (Morris et al, 1994*a*; 1997; present study) resulted in CBE factors that varied from 0.88 to 0.48. In contrast to BSH, major differences were found in the relative distribution of $^{10}B$ in the blood and CNS parenchyma after the administration of BPA at different dose levels. At the highest concentration of $^{10}B$ in the blood (approximately 90 $\mu g\ g^{-1}$), levels of $^{10}B$ in the CNS parenchyma were relatively low, whereas at the lowest concentration of $^{10}B$ in the blood (19 $\mu g\ g^{-1}$) levels of $^{10}B$ in the CNS parenchyma were relatively high at the time of irradiation. These major changes in the biodistribution of $^{10}B$ are the most probable cause of the variations in the calculated CBE factor. At low levels of $^{10}B$ in the blood, the extra luminal dose from the $^{10}B(n,\alpha)^7Li$ reaction is relatively high, whereas at high levels of $^{10}B$ in the blood it is relatively low. Because the extraluminal dose from the $^{10}B(n,\alpha)^7Li$ reaction is not included in the CBE factor calculations, the CBE factor appears to be higher at low $^{10}B$ blood concentrations than at high $^{10}B$ blood concentrations. It should be noted when comparing the two lowest blood $^{10}B$ concentration groups (12 and 19 $\mu g\ g^{-1}$) that the calculated CBE factors were similar. This was in spite of the fact that the blood:CNS $^{10}B$ concentration ratios differed by a factor of approximately 2. However, at these relatively low blood $^{10}B$ levels the overall $^{10}B(n,\alpha)^7$ Li dose (intra- and extraluminal components), expressed as a percentage of the total physical dose delivered to the blood vessel endothelial cell, are similar (approximately 50%).

Owing to the fact that the rate of accumulation/clearance of $^{10}B$ (BPA) from the CNS parenchyma is relatively slow, variation in $^{10}B$ concentration over the time course of irradiation is negligible. This is true in both experimental and clinical studies (Coderre et al, 1996), using BPA as the neutron capture agent. The most salient consideration in translating experimentally derived CBE factors for BPA to the clinical situation is similarity in the $^{10}B$ distribution in the CNS of the animal model and man. At the low dose of BPA (250 mg $kg^{-1}$) currently in use in the BNL clinical trial, $^{10}B$ distribution in the human brain is similar to that in the rat or dog at low blood $^{10}B$ concentrations (Coderre et al, 1992 and unpublished results; Morris et al, 1994*a*).

Supra-additive interactions between photons and fast neutrons or $\alpha$-particles in a mixed irradiation field have been demonstrated in vitro (Murthy et al, 1975; Railton et al, 1975; McNally et al, 1984; 1988). The combined biological effect of two different types of radiation is considered to be supra-additive if it is greater than the two radiations acting independently. In a previous study on the rat spinal cord, using BSH as the neutron capture agent (Morris et al, 1996*a*), the ratio of low LET radiation (gamma rays) to high LET radiation (fast neutrons, $\alpha$-particles, $^7Li$ particles and induced protons) varied from approximately 1:5 to approximately 1:19 as the blood $^{10}B$ concentration was increased from approximately 20 $\mu g\ g^{-1}$ to approximately 120 $\mu g\ g^{-1}$. No evidence of a super-additive effect was found. This finding would tend to preclude super-additivity as a contributory factor in the variation in the value of the CBE factor for BPA found in the present study.

The present findings have obvious clinical implications. The current treatment protocol in the Brookhaven BNCT clinical studies involves a BPA dose of 250 mg $kg^{-1}$ body weight. This delivers blood $^{10}B$ concentrations of 10–15 $\mu g\ g^{-1}$ at the time of treatment. Simulation of the radiation doses delivered to the tumour of a typical glioma patient involved in the Brookhaven study, indicates a distinct advantage in moving to considerably higher blood $^{10}B$ levels than those currently used. For example, at a blood $^{10}B$ concentration of 40 $\mu g\ g^{-1}$, the minimum tumour dose

© Cancer Research Campaign 1997

(i.e. the radiation dose delivered to the tumour at its maximum depth in the brain) can be increased by approximately 50%. A CBE factor of 1.3 was used in the dose calculations. Given the fact that in the current BNL clinical protocol BPA administration involves a 2-h infusion followed by a 45-min gap before the commencement of irradiation, it is unlikely that the CBE factor for the normal CNS will be lower than 1.3 (i.e. the blood:CNS $^{10}B$ concentration ratio is unlikely to exceed 3:1). The experimental data from rat CNS studies suggests that 3 h after the administration of BPA the blood: CNS $^{10}B$ concentration ratio is unlikely to exceed 3:1, even after high doses of BPA. However, the possibility exists that this ratio could vary in future BPA administration protocols designed to maximize tumour uptake of $^{10}B$. The findings of the present study suggest that such variations are unlikely to result in an increase in the CBE factor for BPA, and could result in lower CBE factors at blood:CNS $^{10}B$ concentration ratios in excess of 3:1.

The microdistribution of $^{10}B$ in rats given BPA has recently been analysed in two brain tumour models using ion microscopy (Smith et al, 1996). It was noted that the $^{10}B$ content of local metastatic cells was about half that of viable cells in the adjacent primary tumour. This finding, taken together with the results of the present study, strongly supports the use of considerably higher doses of BPA in future BNCT clinical protocols to optimize the radiation dose to any local metastatic spread.

### CONCLUSIONS

The biodistribution profile of BPA in the normal CNS of the rat changed as levels of $^{10}B$ increased in the blood. The blood:CNS $^{10}B$ concentration ratio decreased progressively with time after BPA administration. It is this ratio, rather than absolute blood $^{10}B$ levels, that determines the magnitude of the CBE factor. The higher the ratio the lower the CBE factor. Data from the present study indicate that, using the current BNCT clinical administration protocol for BPA, the CBE factor is unlikely to increase when higher BPA doses are used. Computer simulations using the BNL clinical treatment planning software indicate that a significant boost in the radiation dose delivered to the tumour at depth in the brain can be achieved by using higher BPA dosages than those in current clinical use.

### ACKNOWLEDGEMENTS

This work was supported by the UK Department of Health. Additional support was provided by the Office of Health and Environmental Research, US Department of Energy, under contract number DE-ACO2-76CH00016.

### REFERENCES

Bloom HJG (1982) Intacranial tumours: Response and resistance to therapeutic endeavours. *Int J Radiat Oncol Biol Phys* **8**: 1083–1113

Calvo W, Hopewell JW, Reinhold HS and Yeung TK (1988) Time and dose-related changes in the white matter of the brain after single doses of X rays. *Br J Radiol* **61**: 1043–1052

Coderre JA, Joel DD, Micca PL, Nawrocky MM and Slatkin DN (1992) Control of intracerebral gliosarcomas in rats by boron neutron capture therapy with p-boronophenylanaline. *Radiat Res* **129**: 290–296

Coderre JA, Makar MS, Micca PL, Nawrocky MM, Joel DD, Slatkin DN and Amols HI (1993) Derivations of the relative biological effectiveness of the high-LET produced during boron neutron capture therapy of the 9L rat gliosarcoma in vitro and in vivo. *Int J Radiat Oncol Biol Phys* **27**: 1121–1129

Coderre JA, Button TM, Micca PL, Fisher CD, Nawrocky MM and Liu HB (1994) Neutron capture therapy of the 9L rat gliosarcoma using the p-boronophenylalanine-fructose complex. *Int J Radiat Oncol Biol Phys* **30**: 643–652

Coderre JA, Morris GM, Micca PL, Fisher CD and Ross GA (1995) Comparative assessment of single-dose and fractionated boron neutron capture therapy. *Radiat Res* **166**: 310–317

Coderre JA, Bergland R, Capala J, Chadha M, Canana AD, Elowitz E, Joel DD, Liu HB and Slatkin DN (1997) Boron neutron capture therapy for glioblastoma multiforme using p-boronophenylalanine and epithermal neutrons: Trial design and early clinical results. *J Neuro-oncol* (in press)

Gutin PH (1992) Radiation therapy for malignant brain tumours: Status and future prospects. In: *Proceedings of the Fifth International Symposium on Neutron Capture Therapy for Cancer*, pp. 98–104. Plenum Press: New York

Hall EJ (1988) *Radiobiology for the Radiologist*. JB Lippincott: Philadelphia.

Hatanaka H, Sweet WH, Sano K and Ellis F (1991) The present status of boron neutron capture therapy for tumours. *Pure Appl Chem* **63**: 373–374

Joel DD, Fairchild RD, Laissue JA, Saraf SK, Kalef-Ezra JA and Slatkin DN (1990) Boron neutron capture therapy of intracerebral rat gliosarcomas. *Proc Natl Acad Sci USA* **87**: 9808–9812

Kitao H (1975). A method for calculating the absorbed dose near interface from $^{10}B(n,\alpha)^{7}Li$ reaction. *Radiat Res* **61**: 304–315

Leibel SA, Scott CB and Loeffler JS (1994) Contemporary approaches to the treatment of malignant gliomas with radiation therapy. *Semin Oncol* **21**: 198–219

Leibel SA and Sheline GE (1987) Radiation therapy for neoplasms of the brain. *J Neurosurg* **66**: 1–22

McNally NJ, DeRond J and Folkard M (1988) Interaction between X ray and α-particle damage in V79 cells. *Int J Radiat Biol* **53**: 917–920

McNally NJ, DeRond J and Hincliffe M (1984) The effect of sequential irradiation with X rays and fast neutrons on the survival of V79 Chinese hamster cells. *Int J Radiat Biol* **45**: 301–310

Morris GM, Coderre JA, Hopewell JW, Micca PL, Nawrocky MM, Liu HB and Bywaters A (1994*a*) Response of the central nervous system to boron neutron capture irradiation: evaluation using a rat spinal cord model. *Radiother Oncol* **32**: 249–255

Morris GM, Coderre JA, Hopewell JW, Micca PL and Rezvani M (1994*b*) Response of rat skin to boron neutron capture therapy with p-boronophenylalanine or borocaptate sodium. *Radiother Oncol* **32**: 144–153

Morris GM, Coderre JA, Whitehouse EM, Micca P and Hopewell JW (1994*c*) Boron neutron capture therapy: a guide to the understanding of the pathogenesis of late radiation damage to the rat spinal cord. *Int J Radiat Oncol Biol Phys* **28**: 1107–1112

Morris GM, Coderre JA, Hopewell JW, Micca PL and Fisher C (1996*a*) Boron neutron capture irradiation of the rat spinal cord: effects of variable doses of borocaptate sodium. *Radiother Oncol* **39**: 253–259

Morris GM, Coderre JA, Bywaters A, Whitehouse E and Hopewell JW (1996*b*) Boron neutron capture irradiation of the rat spinal cord: histopathological evidence of a vascular mediated pathogenesis. *Radiat Res* **146**: 313–320

Morris GM, Coderre JA, Hopewell JW, Rezvani M, Micca PL and Fisher CD (1997) Response of the central nervous system to fractionated boron neutron capture irradiation: studies with borocaptate sodium. *Int J Radiat Biol* **71**: 185–192

Murthy MSS, Madhavanath U, Subrahmanyam P, Rao RS and Reddy NMS (1975) Synergistic effect of simultaneous exposure to $^{60}C$ gamma rays and $^{210}Po$ alpha rays in diploid yeast. *Radiat Res* **63**: 185–190

Nigg DW, Wheeler FJ, Wessol DE, Capala J and Chada M (1997) Radiation physics and treatment planning for boron neutron capture therapy of glioblastoma multiforme. *J Neuro-Oncol* (in press)

Railton R, Lawson RC and Porter D (1975) Interaction of τ-ray and neutron effects on the proliferative capacity of Chinese hamster cells. *Int J Radiat Biol* **27**: 75–82

Rydin RA Deutsch OL and Murray BB (1976) The effect of geometry on capillary wall dose for boron neutron capture therapy. *Phys Med Biol* **21**: 134–138

Smith DR, Chandra S, Coderre JA and Morrison GH (1996) In microscopy imaging of $^{10}B$ from p-boronophenylalanine in a brain tumour model for boron neutron capture therapy. *Cancer Res* **56**: 4302–4306

Wong CS, Poon JK and Hill RP (1993) Re-irradiation tolerance in the rat spinal cord: influence of initial level of damage. *Radiother Oncol* **26**: 132–138

© Cancer Research Campaign 1997